# Genome-Wide Approaches to Unravel the Host Factors Involved in Chikungunya Virus Replication

**DOI:** 10.3389/fmicb.2022.866271

**Published:** 2022-03-24

**Authors:** Atsushi Tanaka, Youichi Suzuki

**Affiliations:** ^1^Division of Research Animal Laboratory and Translational Medicine, Research and Development Center, Osaka Medical and Pharmaceutical University, Takatsuki, Japan; ^2^Department of Microbiology and Infection Control, Faculty of Medicine, Osaka Medical and Pharmaceutical University, Takatsuki, Japan

**Keywords:** Chikungunya virus, host factors, genome-wide screen, replication, antivirals

## Abstract

Chikungunya virus (CHIKV), the causative agent of Chikungunya fever (CHIKVF) that is often characterized by fever, headache, rash, and arthralgia, is transmitted to humans by *Aedes* mosquito bites. Although the mortality rate associated with CHIKV infection is not very high, CHIKVF has been confirmed in more than 40 countries, not only in tropical but also in temperate areas. Therefore, CHIKV is a growing major threat to the public health of the world. However, a specific drug is not available for CHIKV infection. As demonstrated by many studies, the processes completing the replication of CHIKV are assisted by many host factors, whereas it has become clear that the host cell possesses some factors limiting the virus replication. This evidence will provide us with an important clue for the development of pharmacological treatment against CHIKVF. In this review, we briefly summarize cellular molecules participating in the CHIKV infection, particularly focusing on introducing recent genome-wide screen studies that enabled illuminating the virus-host interactions.

## Introduction

Chikungunya virus (CHIKV) is an enveloped RNA virus that causes Chikungunya fever (CHIKF) in humans. CHIKV is classified into the *Alphavirus* genus in the *Togaviridae* family, which is composed of more than 30 recognized viruses (Ahola et al., 2021). Most alphaviruses are transmitted by mosquitoes, and therefore they are also often referred to as arboviruses (arthropod-borne viruses) (Ahola et al., 2021). A CHIKV particle is approximately 70 nm in size and contains a single-stranded, positive-sense RNA genome (approximately 12 kb in length) within an icosahedral capsid structure. Four non-structural (nsP1, nsP2, nsP3, and nsP4) and five structural (C, E3, E2, 6k, and E1) proteins are encoded in the 5′-terminal two-thirds and 3′-terminal one-third portion of the viral genome, respectively (Campion et al., 2015).

Chikungunya virus strains are categorized into Asian, East/Central South African (ECSA), and West African (WA) lineages based on the E1 gene sequence (Weaver and Forrester, 2015). Although CHIKF had been regarded as an endemic disease that caused sporadic epidemics in Africa and Asia, the massive outbreak that started in 2004 in coastal Kenya increased awareness of CHIKV infection and led to its recognition as a re-emerging global disease (Weaver and Forrester, 2015). It is noteworthy that the ECSA lineage having an alanine to valine substitution at position 226 of the E1 protein was shown to play a key role in the spread of CHIKV during the outbreak (Volk et al., 2010). Supporting this, *in vitro* studies revealed that the A226V substitution in E1 enhanced the replication fitness of CHIKV in the *Aedes albopictus* mosquito, which thrives in both tropical and temperate regions (Tsetsarkin et al., 2007; Vazeille et al., 2007; Weaver and Forrester, 2015). However, it has also been demonstrated that E1-A226V was not a sole determinant for the molecular adaptation of the CHIKV ECSA lineage to the *Ae. albopictus* cell (Wikan et al., 2012; Suzuki et al., 2021).

Clinical symptoms of CHIKVF generally include a sudden onset of fever, myalgia, and arthralgia after an incubation period of 2–6 days (Couderc and Lecuit, 2015). However, it has been reported that around 15% of infected people show no symptoms (Lemant et al., 2008). Arthralgia occurs symmetrically in the extremities, especially in the wrists, ankles, and toes, frequently accompanied by skin rash, headache, myalgia, lymphadenopathy, and nausea (Couderc and Lecuit, 2015). In the acute phase, the viral RNA per milliliter of blood reaches up to 10^9^ copies, and the high level of viremia was shown to be often correlated with the severity of the medical condition (Parola et al., 2006; Staikowsky et al., 2009). Although these symptoms are mostly resolved within 10 days, in some patients, polyarthritis develops, and joint pain persists for months to years (Couderc and Lecuit, 2015). The chronic disease is not likely to be a persistent infection of CHIKV; the mechanism that leads to the chronicity of these joint symptoms remains unclear (Schwartz and Albert, 2010). Mortality associated with CHIKV infection is not high (Josseran et al., 2006), whereas the risk of severe disease increases in young children, elderly people, and individuals undergoing the treatment for hypertension, diabetes, or heart disease, in which encephalitis, cardiovascular disorder, renal failure, hepatitis, and myocarditis may occur (Schwartz and Albert, 2010).

Although CHIKVF is generally considered a non-fatal self-limiting disease, CHIKV infection, particularly that associated with prolonged arthralgia, has a negative impact on the health-related quality of life of patients (Soumahoro et al., 2009; Staikowsky et al., 2009; Kumar et al., 2021). Therefore, the development of safe and effective antiviral drugs is required for the treatment of CHIKV infection (Burt et al., 2017). To date, many small molecule inhibitors against CHIKV have been developed, and their anti-CHIKV activities have been validated in *in vitro* experiments. However, since the cellular proteins targeted by the inhibitors (such as kinases and chaperone molecules) are often involved in critical biological activities of the host, the application of candidate inhibitors to the treatment of CHIKV-infected individuals remains an obstacle (Haese et al., 2022). In this respect, a comprehensive understanding of the molecular interactions between the virus and host cell should provide helpful insights into the more promising druggable target(s) for the development of anti-CHIKV agents. In this review, we focus on several cellular factors promoting or restricting CHIKV infection identified by genome-wide screen approaches.

## Survey of Cellular Proteins Involved in the Attachment of Chikungunya Virus

When a mosquito infected with a mosquito-borne virus such as CHIKV bites a target host, the virus is injected into the small blood vessels and capillaries of the animal along with the mosquito saliva, which acts as an anti-vasoconstrictor and an anticoagulant (Ribeiro and Francischetti, 2002). Hence, blood cells are considered the primary target cells for CHIKV infection (Her et al., 2010). However, many other types of cells have been reported to be susceptible to CHIKV (Wikan et al., 2012; Roberts et al., 2017).

**FIGURE 1:**
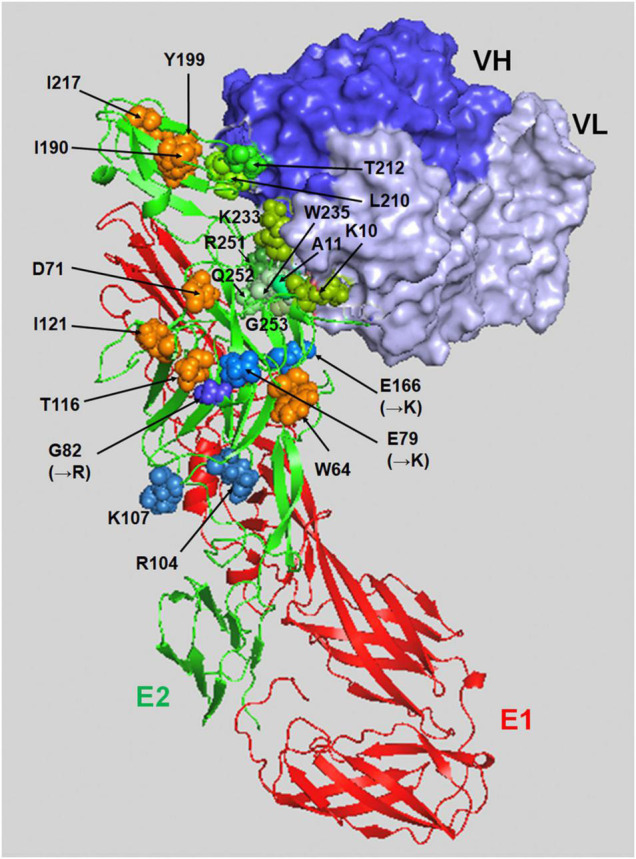
A binding model of the E2-E1 heterodimer and NAb CHE19 on the CHIKV Thai#16856 spike. The binding of CHE19 Fab fragment (surface drawings, blue: the heavy chain, light blue: light chain) on the E2-E1 heterodimer (ribbon drawings reconstructed using PDB ID 3N42, red: the E1 glycoprotein, green: the E2 glycoprotein) is shown. The residues shown as green spheres (K10, A11, L210, T212, K233, W235, R251, Q252, and G253) are in contact within 4 Å of the heavy atoms of the NAb CHE19 paratope. The optimal binding sites of Mxra8-Fc are shown as orange spheres (W64, D71, T116, I121, I190, Y199, and I217). The residues shown as blue spheres are as follows; R104 and K107 in the predicted E2 amino acids for HS binding, and the residues substituted to the positively charged ones in 181/25 vaccine strain [G82(→R)] and mutant [E79(→K) and E166(→K)] in mutant CHIKV. Those positively charged residues are responsible for HS binding. This image was visualized using PyMOL software.

The replication of CHIKV in humans begins with the attachment of virus particles to the surface of the target cell. The CHIKV virion is enveloped by the lipid bilayer membrane, which contains 80 viral envelope spikes trimerized with the heterodimer of E1-E2 glycoproteins (Simizu et al., 1984; Jose et al., 2009; Voss et al., 2010; Yap et al., 2017). E1 is a class II pH-triggered membrane fusion protein that is positioned at the base of the spike, and the top of E1 is covered by a protector protein, E2, which is located on the distal end of the spike (Li et al., 2010; Modis, 2013). Thus far, several cell surface proteins have been implicated as attachment receptors for CHIKV (Schnierle, 2019). A recent CRISPR-Cas9-based genome-wide screen revealed that the cell adhesion molecule Mxra8 (also known as DICAM, ASP, or Limitrin) is a receptor molecule mediating the entry of multiple alphaviruses, notably CHIKV (Zhang et al., 2018). Mxra8 is reported to be involved in cell-cell adhesion through a heterophilic interaction with αVβ3 integrin and associated with osteoclast differentiation and angiogenesis (Jung et al., 2012; Han et al., 2013). Cryo-electron microscopy (cryo-EM) and mutagenesis studies revealed that Mxra8 binds by wedging into a cleft created by two adjacent CHIKV E2-E1 heterodimers in one trimeric spike and engaging a neighboring spike; they also showed that Mxra8 binds to a surface-exposed region across the A and B domains of CHIKV E2, with speculated residues W64, D71, T116, and I121 in the A domain and I190, Y199, and I217 in the B domain (as shown in Figure 1), which emerged as essential for optimal Mxra8-Fc binding. Of interest is that CD147, identified as a novel cellular protein involved in CHIKV entry, was found to have a structural topology similar to that of Mxra8 in its two immunoglobulin-like domains (Caluwé et al., 2021). More importantly, human monoclonal antibodies competing for the interaction of CHIKV E2 glycoprotein and Mxra8 were shown to be protective against CHIKV infection in mice (Zhang et al., 2019; Powell et al., 2020), holding promise as a therapeutic antibody drug for the treatment of CHIKF. However, it is unclear whether Mxra8 is a necessary and sufficient receptor for CHIKV infection since some CHIKV-susceptible cell lines do not express Mxra8, and CHIKV is still able to infect the Mxra8 knockout mice.

It is well known that cell surface glycosaminoglycans (GAGs) bind to various bioactive proteins such as cell growth factors, cytokines, chemokines, enzymes, and protease inhibitors to regulate their activities (Sarrazin et al., 2011). In addition, they are also reported as the primary attachment factors, co-receptors, or the molecule that concentrates virion on the cell surface before entry for various virus infections (Rostand and Esko, 1997; Shukla et al., 1999; Aquino and Park, 2016). GAGs are unbranched, high-molecular-weight polysaccharides that contain repeating disaccharide units of N-acetylglucosamine (GlcNAc) and D-glucuronic acid (GlcA) in the heparan sulfate (HS) backbone and disaccharide units of GalNAc and GlcA in the chondroitin sulfate (CS) backbone. GAGs attach to specific sites on the core proteins, generating proteoglycans (Häcker et al., 2005). Several studies using a live attenuated vaccine strain (181/25) and mutant CHIKV revealed that the viral determinant responsible for GAG dependency was in the E2 protein (Levitt et al., 1986; Silva et al., 2013; Gardner et al., 2014; Weber et al., 2017). Point mutations within the E2 protein (e.g., E79K, G82R, or E166K, shown in Figure 1) have been found in attenuated vaccine strains and in mutant viruses that exhibited enhanced GAG dependency but reduced *in vivo* pathogenicity (Gardner et al., 2014). In the attenuated CHIKV strain 181/25, the substitution of a residue at 82 (arginine to glycine) in the E2 glycoprotein showed a higher titer in the spleen and serum of mice at early times after inoculation (Ashbrook et al., 2014). Our previous genome-wide approach using knockout HAP1 cell libraries generated by a piggyBac-transposon-based exon-trapping vector found that the authentic clinical isolate CHIKV also utilizes the cell surface GAGs for entry to the target cell (Tanaka et al., 2017). We showed that a clinical CHIKV isolate (Thai#16856 strain) and prototype CHIKV (Ross strain), which contained the 79E, 82G, and 166E in E2, had a higher affinity to HS and that the N-sulfated HS was the minimum structure required for efficient CHIKV binding and infection on HAP1 cells (Tanaka et al., 2017). In addition, although the CS, another GAG, has been reported to be associated with viral infection, including CHIKV, CS may participate at later steps of CHIKV replication after virion binding (Banfield et al., 1995; Kato et al., 2010; Kim et al., 2011; Jinno-Oue et al., 2013; Silva et al., 2013; Zhang et al., 2013; Tanaka et al., 2017). Interestingly, previous studies have shown that pentosan polysulfate, an HS-like molecule, was capable of reducing the viral titer of alphaviruses including CHIKV *in vitro* and *in vivo*, indicating the potential therapeutic use of the GAG mimetic for the treatment of CHIKV infection in humans (Herrero et al., 2015; Supramaniam et al., 2018).

## Entry and Membrane Fusion Process

**FIGURE 2:**
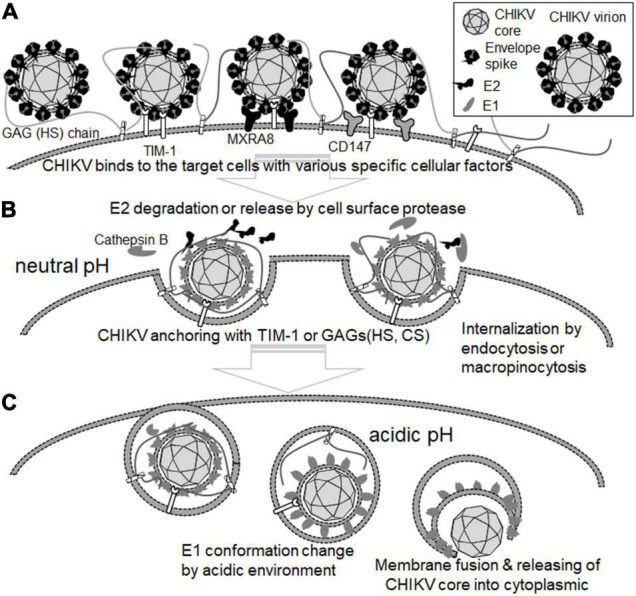
Role of cellular proteins in the attachment and entry steps of CHIKV. **(A)** CHIKV virion binds to the target cells with cell surface molecules, including HS-proteoglycan, Mxra8, and CD147. **(B)** E2 is degraded and eliminated from CHIKV virion by cell surface protease such as cathepsin B at the neutral pH. CHIKV virions bearing no E2 are anchored by TIM-1, HS-/CS-proteoglycan, and internalized by endocytosis or micropinocytosis. **(C)** The conformation of the E1 protein is changed at the acidic pH. Then, the membrane of CHIKV virions and the target cell membrane are fused by the E1 protein, resulting in the release of the CHIKV core into the cytoplasm of the target cell.

Endocytosis is one of the major machineries for the entry of many viruses into target cells (Smith and Helenius, 2004). In the case of CHIKV infection, clathrin-dependent endocytosis is considered to be the main pathway for virion uptake into cells (Bernard et al., 2010; Kielian et al., 2010), although micropinocytosis is also reported as a route of CHIKV entry (Lee et al., 2019; Izumida et al., 2020). After internalization of the virion, membrane fusion between CHIKV and the cell occurs within the endosomal compartment, which is triggered by the low pH environment of the endosomes. This acidic pH then induces the dissociation of E1 from the E1/E2 glycoprotein dimer of CHIKV, followed by the penetration of E1 into the cell membrane (Kielian et al., 2010). Although the details of the structural change of E2 remain unclear, the R104 and K107 of E2 were shown to induce conformational change, and these residues were expected to configure the HS-binding pocket (Figure 1). Additionally, these two positive-charge residues forming the HS-binding sequence motif (XBXXBX, where B is a basic residue) were conserved in all CHIKV strains (Sahoo and Chowdary, 2019). Recently, we revealed that the E2 proteins of cell-bound CHIKV were easily lost during viral internalization, which was also observed in the cells that inhibited the endosome acidification *via* bafilomycin A1 treatment, suggesting that part of the conformational changes in E2 occurs before endosome acidification (Tumkosit et al., 2019). In addition, a CHIKV-neutralizing monoclonal antibody (NAb), CHE19, recognizes the E2 protein (Figure 1), which inhibits viral membrane fusion by stabilizing the E2-E1 heterodimer instead of E3, blocking the elimination of E2 (Tumkosit et al., 2020). CHIKV E2 may promptly suffer degradation by some type of existing protease cell membrane, as reported in other enveloped viruses (Lu et al., 1996; Abe et al., 2013; Bertram et al., 2013; Park et al., 2016). Indeed, it was recently shown that cathepsin B protease facilitated CHIKV envelope-mediated infection *via* endocytosis or macropinocytosis (Izumida et al., 2020). Thus, the binding position of neutralizing antibody CHE19 may be a target site for the protease that digests the E2 of the CHIKV virion after binding. Given that cell surface proteases dissociate the E2 of the virion bound to the target cell during the CHIKV entry, it is plausible that GAGs or the T-cell immunoglobulin and mucin domain 1 (TIM-1) (Kirui et al., 2021) may be used as an anchoring factor for tethering the E2-lacking virion on the cell surface in the endosome (Figure 2).

A genome-wide screen study employing small interfering RNA (siRNA) identified fuzzy homolog (FUZ) and TSPAN9 as cellular proteins that promoted the entry process of CHIKV (Ooi et al., 2013). Depletions of FUZ and TSPAN9 showed a significant reduction of CHIKV in human cells, and the FUZ depletion was likely to hamper the internalization step of another alphavirus, Semliki Forest virus (SFV) (Ooi et al., 2013). In contrast, the silencing of TSPAN9 inhibited the intracellular membrane fusion of alphavirus in endosomes, and it was characteristic of the viruses that fused in early endosomes, such as CHIKV (Ooi et al., 2013; Duijl-Richter et al., 2015; Stiles and Kielian, 2016). Additionally, the siRNA screen study by Ooi et al. revealed that Archain 1 (ARCN1), a subunit of the COPI coatomer complex, promoted the binding of alphaviruses SFV and Sindbis virus (SINV), therefore providing new insight into the involvement of cellular factors in the early events of alphavirus infection including CHIKV infection (Ooi et al., 2013).

## Application of a Genome-Wide Screen to Investigate the Cellular Factors Essential to Intracellular Chikungunya Virus Replication

After entry into the target, an open reading frame (ORF) encoding nsP1–4 is first translated from the viral RNA released into the cytoplasm, which yields precursors of the non-structural protein. It has been well demonstrated that the majority of CHIKV isolates possess an opal stop codon (UGA) between the nsP3 and nsP4 genes that produces an nsP123 precursor; on the other hand, a full-length nsP1234 polyprotein is generated by the readthrough of the opal stop codon (Li and Rice, 1993; Jones et al., 2017). The nsP4 that is initially cleaved from the nsP1234 precursor functions as an RNA-dependent RNA polymerase (RdRp), together with nsP123, for the synthesis of negative-sense RNA, which, in turn, serves as a template for the amplification of full-length (49S) positive-sense RNA (Schwartz and Albert, 2010; Ahola et al., 2021). In contrast to the non-structural protein expression, structural protein is translated from the subgenomic (26S) RNA that is transcribed under the internal promoter sequence between two ORFs of non-structural and structural proteins (Ahola et al., 2021). The capsid (C) protein, which is cleaved from a structural protein precursor by its autoprotease activity, associates with 49S genomic RNA to form a nucleocapsid core (Schwartz and Albert, 2010; Ahola et al., 2021). Concurrently, the rest of the structural proteins containing E glycoproteins are processed and matured through the translocation from the endoplasmic reticulum (ER) to Golgi compartments and assembled with a nucleocapsid below the plasma membrane. Eventually, the mature virion egresses from the infected cell *via* budding (Schwartz and Albert, 2010).

nsP3 is an accessory protein necessary for the nsP4’s RNA polymerase activity and has been shown to possess ADP-ribosylhydrolase activity in its N-terminal domain, whereas the C-terminal domain is hypervariable (Eckei et al., 2017; McPherson et al., 2017). In a recent study, CRISPR-Cas9-based genetic screening found four-and-a-half LIM domain protein 1 (FHL1) as a host factor essential for CHIKV replication (Meertens et al., 2019). FHL1, a member of the FHL family of proteins that are characterized by the existence of LIM domains, is predominantly expressed in skeletal muscle and is thought to be involved in muscle development and maintenance (Shathasivam et al., 2010). In CHIKV-infected cells, FHL1 interacted with the hypervariable domain of nsP3 and appeared to play a critical role in viral RNA synthesis (Meertens et al., 2019). It was also demonstrated that FHL1-deficient mice were less susceptible to CHIKV infection, and more importantly, virus replication was greatly impaired in fibroblasts and myoblasts derived from Emery-Dreifuss muscular dystrophy (EDMD) patients, in which the FHL1 gene was mutated (Gueneau et al., 2009; Shathasivam et al., 2010). This genome-wide screening study demonstrates that FHL1 is a major determinant for the susceptibility of humans to CHIKV. In addition, since the expression of FHL1 is mainly found in skeletal muscle cells, the molecular interaction between nsP3 and FHL1 would influence the progression of arthritis in CHIKV-infected patients. Therefore, this host-virus interaction could be a promising target for the development of antivirals against CHIKV disease (Meertens et al., 2019).

A study using a set of siRNA libraries targeting cellular factors involved in membrane trafficking revealed critical roles of endosomal sorting complexes required for transport (ESCRT) proteins in the intracellular replication of CHIKV (Torii et al., 2020). ESCRT, originally discovered in yeast cells, are a network of the cytoplasmic protein complex, which has been demonstrated to regulate cellular membrane fission events, including the multivesicular body (MVB) formation and cytokinesis (Henne et al., 2011; Morita, 2012). One impact of the ESCRT system in virology is that many enveloped viruses, such as human immunodeficiency virus type 1 (HIV-1), exploit the ESCRT proteins for their replication (Meng and Lever, 2021). As for CHIKV replication, siRNA-mediated depletion of 13 ESCRT genes markedly reduced the level of virus replication in HEK293T cells. Interestingly, some of the ESCRT factors were found to be required for CHIKV RNA synthesis and the post-translation step, which was presumably at the extracellular release step of the virion, as reported in HIV-1 (Torii et al., 2020). Hence, this siRNA screen study sheds light on the important role of the ESCRT pathway in the biology of CHIKV.

**FIGURE 3:**
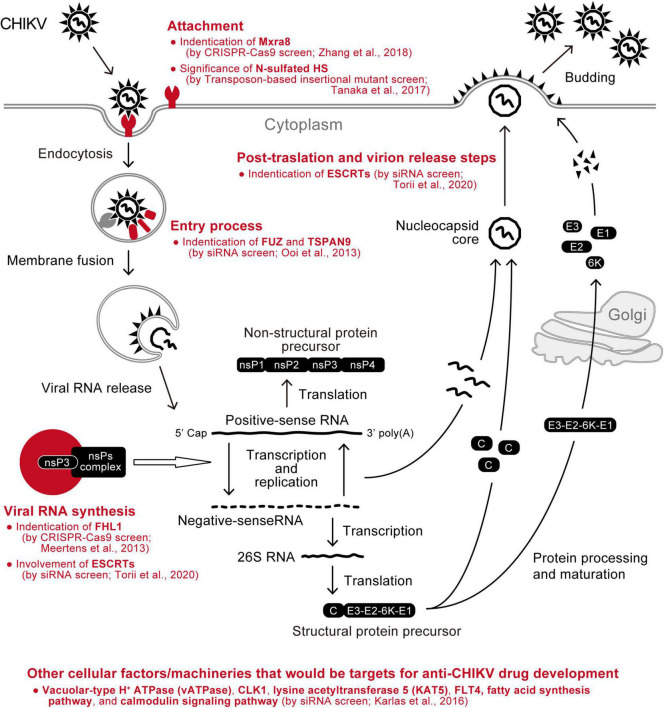
Summary of CHIKV-related cellular factors identified by genetic screens. Cellular factors that have been found as enhancers of CHIKV replication using comprehensive screening approaches (red) are depicted in the schematic of the virus replication cycle.

The genome-wide loss-of-function screen approach has also been employed to seek a druggable cellular target suitable for inhibiting CHIKV replication. Karlas et al. performed transfection of a large set of siRNA libraries using HEK-293 cells, followed by infection with green fluorescent protein (GFP)-expressing CHIKV, and identified 156 enhancing and 41 inhibitory genes for virus replication (Karlas et al., 2016). Then, by querying the enhancer hits against the databases of drugs whose target molecules have been experimentally proven, 52 chemical compounds were selected as antiviral candidates against CHIKV, and 20 of them indeed inhibited CHIKV *in vitro*. Furthermore, three drugs targeting the fatty acid synthesis pathway, calmodulin signaling pathway, or fms-related tyrosine kinase 4, all of which were identified as cellular enhancers for CHIKV infection using an siRNA library screen, significantly reduced virus replication in C57BL/6 mice (Karlas et al., 2016). Therefore, this is a proof-of-concept study demonstrating that the genome-wide screen is beneficial for a comprehensive survey of potential antiviral agents against CHIKV (Figure 3).

## Functional cDNA Expression Cloning to Find Cellular Inhibitory Factors Against Chikungunya Virus

Generally, the loss-of-function genetic screen using the siRNA and CRISPR-Cas9 system provides insights into the dependencies of the virus on host factors and machinery, whereas the gain-of-function screen, which ectopically expresses a certain set of functional genes, is able to identify cellular factors that limit virus replication. Particularly, it has been demonstrated that the gain-of-function screen using a cDNA library is a powerful approach in a comprehensive study of host antiviral mechanisms such as interferon (IFN)-stimulated genes (Schoggins et al., 2011; Kane et al., 2016; Schoggins, 2019). Recently, we applied an expression-cloning screen using the cDNA library, which was generated from type I IFN-treated human cells, to CHIKV infection (Sakaguchi et al., 2020). The African green monkey–derived Vero cell is highly permissive of CHIKV and exhibits a massive cytopathic effect with the infection (Schwartz and Albert, 2010). However, when Vero cells were transduced with a pool of HIV vectors carrying the IFN-related cDNA library and subsequently subjected to a challenge infection with CHIKV, many cells that survived the viral infection were obtained. Then a long-read sequencing analysis using the MinION sequencer (Clarke et al., 2009) showed that cDNAs encoding three different mitochondrial proteins (TOM7, S100A16, and ECI1 lacking the N-terminal 59 amino acids) were introduced to the CHIKV-resistant cells. The inhibitory activities of these cellular factors were confirmed by an over-expression experiment using human Huh7 cells (Sakaguchi et al., 2020). One plausible molecular mechanism by which these cellular factors limit the CHIKV replication would be that the expression of TOM7 and S100A16 reinforced the function of mitochondria, resulting in the up-modulation of cellular innate immune response (Kim et al., 2018). Meanwhile, the expression of the N-terminally deleted ECl1 may function as a dominant-negative mutant for the lipid metabolism, which is usually catalyzed by wild-type ECI1 in the mitochondria and shown to be required for the replication of RNA viruses (Takahashi et al., 2007; Rasmussen et al., 2011). Although endogenous expressions of these mitochondria-related proteins were not changed in human cells upon IFN treatment (Sakaguchi et al., 2020), this study illustrates the usefulness of the gain-of-function cDNA library screening approach in the search for cellular inhibitors against CHIKV.

## Concluding Remarks

As seen in many human pathogenic viruses, CHIKV hijacks the host machinery to create a favorable environment for virus replication (Wong and Chu, 2018). On the other hand, the host cells harbor countermeasure mechanisms that restrict CHIKV replication (Schwartz and Albert, 2010; Schneider et al., 2014). Understanding these virus-host relationships, which are key factors influencing disease pathogenesis and progression, should reveal the Achilles’ heel of CHIKV and be a basis for the future development of an anti-CHIKV drug. In particular, antiviral agents targeting the molecular interactions between CHIKV and cellular factors hold the promise of avoiding the emergence of resistant viruses (Wong and Chu, 2018). From this viewpoint, recent advances in genome-wide screening technologies could provide a complete molecular picture of the cellular environments where CHIKV replicates in the near future (Ramage and Cherry, 2014). In this review, we summarized the CHIKV-related host factors that have been identified by several genome-wide screen studies (Figure 3). Notwithstanding, it will be important to determine precisely whether the host factors identified are necessary for CHIKV infection and pathogenesis.

## Author Contributions

AT and YS contributed to the conceptualization, writing, review, and editing of this manuscript. Both authors have read and agreed to the submission of the manuscript.

## Conflict of Interest

The authors declare that the research was conducted in the absence of any commercial or financial relationships that could be construed as a potential conflict of interest.

## Publisher’s Note

All claims expressed in this article are solely those of the authors and do not necessarily represent those of their affiliated organizations, or those of the publisher, the editors and the reviewers. Any product that may be evaluated in this article, or claim that may be made by its manufacturer, is not guaranteed or endorsed by the publisher.
